# Development and results of a novel emergency medicine residency research immersion program

**DOI:** 10.1017/cts.2024.626

**Published:** 2024-10-22

**Authors:** Kaitlin Ray, Catherine Burger, Alexander T. Clark, Emily K. Pauw, Wesley H. Self, Jesse O. Wrenn, William B. Stubblefield, Jin H. Han, Michael J. Ward

**Affiliations:** 1 Department of Emergency Medicine, Vanderbilt University Medical Center, Nashville, TN, USA; 2 Department of Emergency Medicine, Division of Critical Care, University of Michigan Hospital, Ann Arbor, MI, USA; 3 Vanderbilt Institute for Clinical Translation Research, Vanderbilt University Medical Center, Nashville, TN, USA; 4 Geriatric Research, Education, and Clinical Center (GRECC), Tennessee Valley Healthcare System, Nashville, TN, USA; 5 Department of Biomedical Informatics, Vanderbilt University Medical Center, Nashville, TN, USA

**Keywords:** Emergency medicine, training, career development, evaluation, program development

## Abstract

Creating a sustainable residency research program is necessary to develop a sustainable research pipeline, as highlighted by the recent Society for Academic Emergency Medicine 2024 Consensus Conference. We sought to describe the implementation of a novel, immersive research program for first-year emergency medicine residents. We describe the curriculum development, rationale, implementation process, and lessons learned from the implementation of a year-long research curriculum for first-year residents. We further evaluated resident perception of confidence in research methodology, interest in research, and the importance of their research experience through a 32-item survey. In two cohorts, 25 first-year residents completed the program. All residents met their scholarly project requirements by the end of their first year. Two conference abstracts and one peer-reviewed publication were accepted for publication, and one is currently under review. Survey responses indicated that there was an increase in residents’ perceived confidence in research methodology, but this was limited by the small sample size. In summary, this novel resident research curriculum demonstrated a standardized, reproducible, and sustainable approach to provide residents with an immersive research program.

## Introduction

### Need for innovation

Barriers to emergency medicine resident research exist at the individual, program, and departmental levels as recognized by the recent Society for Academic Emergency Medicine (SAEM) 2024 Consensus Conference, *Creating a Diverse and Sustainable Emergency Medicine Investigator Pathway* [1]. These barriers not only present challenges for the development of a diverse and sustainable research workforce capable of tackling health care’s most complex problems, but also specifically limit graduates from emergency medicine residency programs and their abilities to understand, interpret, and apply research findings in their clinical practice.

Given the demands of clinical residency, residents have difficulty completing the scholarship requirement from the Accreditation Council for Graduate Medical Education (ACGME). Barriers to scholarly projects include lack of interest, time, mentoring, support, and skills [2]. Within our emergency medicine program, we found that residents often wait until the final year of residency before conducting a project, limiting the quality of such a project and the opportunity for exposure to research as a potential career path. Further, the administrative burden on the part of residency leadership teams to track the completion of such projects can be quite burdensome. To address these challenges, the Department of Emergency Medicine Resident Leadership Team and Research Division collaborated to develop and implement a novel research program for incoming emergency medicine first-year residents to provide systematic research instruction and research project immersion throughout the first year of residency. We describe this novel, immersive one-year program as a potentially sustainable model for resident research regardless of clinical specialty.

## Background

Presently, the ACGME mandates each emergency medicine resident must participate in scholarship by the time of graduation from their residency program [3]. Specifically, it states “the curriculum must advance the residents” knowledge of the basic principles of research, including how research is conducted, evaluated, explained to patients, and applied to patient care [3]. This may include peer-reviewed publications, non-peer-reviewed work including online publications and podcasts, textbook chapters, and/or conference presentations at the local, regional, or national level [3]. Despite this guideline, the implementation, interpretation, and quality of the scholarly project requirement varies considerably among programs [4]. These challenges limit the development of sustainable, successful resident scholarship and are recognized as challenges for residency programs [5]. To enhance and advance emergency medicine scholarship, Pillow, et al. proposed a framework for scholarly projects suggesting that scholarship should: (1) involve a structured process; (2) generate outcomes; (3) be disseminated; and (4) be peer reviewed [6].

## Methods

### Objective

There were two objectives. First, we sought to advance the quality of the resident scholarly activity through implementation of a sustainable immersive research program for first-year residents. Second, we sought to assess residents’ confidence in research methodology, interest in research, and perceived importance of research experience following this program.

### Development process

This voluntary, year-long program was developed with the Vanderbilt University Medical Center (VUMC) Emergency Medicine Research Division, led by a senior emergency medicine resident, and mentored by content experts and faculty in the Research Division. The VUMC emergency medicine residency is a three-year program in Nashville, Tennessee, USA, that matches 13 residents annually. Representative of other emergency medicine residency programs, residents frequently complete case reports, textbook chapters, and non-peer-reviewed publications, typically toward the end of their three-year residency. Infrequently, residents completed a research project and subsequent peer-reviewed manuscript.

We developed and implemented this program for each cohort of incoming first-year residents because we rationalized that earlier exposure to research would provide residents with a better understanding of how to conduct research and allow residents to decide whether research might be a potential career path for them. Furthermore, exposing them to a group-wide project allows them to work as a cohort before clinical demands increase, which would make it difficult to meet and conduct such a shared project.

The year-long curriculum for each cohort consists of five phases and four, one-hour didactics. The five phases included: (1) preparation; (2) study design and research question; (3) data collection; (4) analysis; and (5) scientific communication. During the preparation phase that occurred prior to first-year resident arrival, a senior resident leader was identified to lead the subsequent resident research project. Each senior resident worked directly with the faculty mentors to propose, develop, and finalize the research question, draft the research protocol, and submit it for institutional review board (IRB) approval. Two faculty (MJW and JH) actively mentored the senior resident in all five phases of the study, met with the senior resident to prepare for and attend didactic sessions, provided additional supervision for data collection, conducted the analysis, and reviewed and edited manuscript drafts. However, the senior resident served as the primary interface with the first-year residents, led all training sessions, and was the first individual to answer questions and review drafts.

Didactic sessions occurred following Tuesday resident conferences to maximize availability and attendance. These four, one-hour didactic sessions corresponded with phases 2-5, study design and research question, data collection, analysis, and scientific communication. All didactics were led by the senior resident with at least one faculty mentor in attendance. Talks included an application to the existing project along with a question-and-answer session.

In the study design and research question phase, first-year residents were exposed to the PICOT (patient, intervention, comparison, outcome, time) elements of a research question and elements of study design through didactic training on the clinical topic. They were further asked to complete the necessary regulatory training. In the data collection phase, study instruments (e.g., REDCap) were developed and introduced for each project. Through the didactic session, residents were taught by the senior resident about elements of data collection, potential limitations, and strategies to mitigate weaknesses in chart review [7]. Residents were further taught how to conduct chart reviews using electronic case report forms for data collection along with good data management practices by developing and maintaining data dictionaries. Data collection was piloted and randomly sampled for quality review to ensure accurate data collection. Official data collection was completed and further sampled to ensure high-quality data collection.

Following data collection, the analysis phase began, and through the didactic session, residents were taught principles of biostatistics used in the project, including descriptive and multivariable models. Data were cleaned and evaluated for missingness, outliers, and nonsensical responses (e.g., negative durations). Study results were presented in the scientific communication phase and a didactic session on preparing an abstract and manuscript was presented, and this coincided with a facilitated discussion with the resident team on the study results and implications of the findings. Sections of the manuscript were then divided into the following sections: journal requirements, abstract, introduction, methods, results, and discussion. Residents were then assigned to draft each section. A national conference abstract and peer-reviewed manuscript were planned for each class and every resident who met authorship criteria was included. During this phase, the senior resident was responsible for assembling each of the sections, editing the overall product, assembling conference and journal submissions, and working with faculty mentors.

### Evaluation

To evaluate the impact of the resident research program, residents from both cohorts were asked to complete an anonymous survey before and after the year-long course. This was kept confidential so that residents could provide candid responses to the questions. The survey assessed confidence in research methodology, experience, and interest in pursuing research during and after residency using a 5-point Likert scale. The survey was distributed via REDCap questionnaires on secure email accounts [11,12]. Mean differences with their 95% confidence interval (95%CI) were calculated and pre- and post-means were compared using a student’s *T*-test.

## Results

### The implementation phase

The first cohort started in the 2022–2023 academic year. We found that sufficient time was necessary (approximately 3–6 months prior to the arrival of each resident class) for co-directors and the senior resident leader to refine the research question, obtain IRB approval, design the REDCap database, set first-year resident expectations, and to create didactic talks for this novel program. We conducted an open solicitation of rising senior residents for potential research questions while identifying residents interested in leading the program. We prioritized research questions that required chart review of a clinical problem to facilitate learning for new residents while gaining familiarity with the electronic health record. Following the first cohort, previous program’s lectures were updated by the senior resident to reflect the current project and any new regulatory requirements or academic content.

Following arrival, first-year residents were involved in every step of the study including finalizing the research question, piloting the data collection forms, and identifying data collection challenges. While there were four planned didactics, we found that each cohort required between one and two additional meetings for data collection training. To maximize resident availability, lectures took place in person on protected residency conference days and were recorded for review for those unable to attend. Finally, we found that the presentation of the results during the scientific communication phase served as an outline for the discussion section.

Existing departmental and research division resources were used to support the program including faculty time and regulatory support. All data management and biostatistics were completed by the mentoring faculty and have not yet required external biostatistical support. We estimated that following the initial startup of the program, this required an average of approximately 2–4 hours of faculty time per month over the course of the academic year.

### Outcomes

Starting with the first cohort in July 2022, we have completed two cohorts in 2022–2023 (year 1) and the 2023–2024 (year 2) academic years, and a third cohort is in process. There was a total of 26 residents, 25 of whom completed the first year of residency and subsequently the research program. Description of the two cohorts can be seen in Table 1 and include 22 (88%) with prior research experience, and among these with a mean (standard deviation) of 3.0 (2.3) years of experience. Among both cohorts, 5 (20%) had an additional graduate degree, and 11 (44%) previously took a formal course on statistical analysis. The year 1 cohort produced a conference abstract that was accepted as a lightning oral abstract presentation at SAEM’s 2024 annual meeting [8] and a peer-reviewed publication [9]. The year 2 cohort had a poster presentation accepted at the American College of Emergency Physicians 2024 Research Forum [10] and a peer-reviewed manuscript is currently under review. Both cohorts have met the ACGME scholarly activity requirement by the completion of their first year of residency.

**Table 1.** Participant demographics from both first-year resident cohorts

	Year 1 Cohort (2022–2023) *N* = 12		Year 2 Cohort (2023–2024) *N* = 13		Overall	
Variable	N	%	N	%	N	%
Prior research experience	10	83%	12	92%	22	88%
Type of Research Experience						
Clinical	6		7		13	
Basic Science	3		2		5	
Both	1		3		4	
Years of research experience, mean, SD	3.4	2.9	2.6	1.6	3.0	2.3
Additional graduate degree	3	25%	2	15%	5	20%
Prior formal course on statistical analysis	6	50%	5	38%	11	44%

Of the 25 residents, 25 (100%) and 21 (84%) responded to the pre- and post-project survey, respectively. Survey responses using a five-point Likert scale indicated that upon completion of this novel research curriculum, there was an increase in four out of 21 elements of residents’ perceived confidence in research methodology queried (Table 2). These included confidence in recognizing the stages of clinical and translational research (pre-post difference 0.68, 95% confidence interval [CI] 0.12–1.24), identifying approaches to enhance the transparency, rigor, and reproducibility of the research project (pre-post difference 0.61, 95%CI 0.02–1.19), listing resources for study design and analysis (pre-post difference 0.57, 95%CI 0.00–1.13), and listing venues to present research (pre-post difference 0.72, 95%CI 0.07–1.38). There was no significant change in confidence in research skills, likelihood to pursue research during and after residency, and perceived importance of research experience for their residency training.

**Table 2.** Survey questions to assess confidence in research methodology, enjoyment of research, interest in pursuing research during and after residency, and the perception of research importance using a five-point Likert scale

Survey Questions	Pre-Curriculum Survey Response (*n* = 25)	Post-Curriculum Survey Response (*n* = 21)	Difference (95%CI)
**Confidence in Research Knowledge – How confident are you in your ability to…**			
appraise the quality of a scientific journal article?	2.48	2.81	0.33 (−-0.19, 0.85)
to define research?	3.00	3.24	0.24 (−0.25, 0.73)
identify various types of research?	2.88	3.29	0.41 (−0.08, 0.89)
recognize stages of clinical/translational research?	2.32	3.00	0.68 (0.12, 1.24)*
to list different types of epidemiology studies?	2.17	2.57	0.40 (−0.19, 1.00)
to define key characteristics and limitations of cross-sectional studies?	2.56	2.86	0.30 (−0.30, 0.90)
define key characteristics and limitations of cohort studies?	2.52	2.86	0.34 (−0.25, 0.93)
**Confidence in Research Skills - How confident are you in your ability to…**			
identify an appropriate research mentor and to develop a research project?	2.56	2.67	0.11 (−0.52, 0.74)
identify steps of the scientific method?	3.24	3.43	0.19 (−0.39, 0.77)
use PubMed for literature searches?	3.32	3.52	0.20 (−0.46, 0.87)
use Google Scholar for literature searches?	3.16	3.43	0.27 (−0.40, 0.93)
identify resources for conducting an effective literature search?	3.20	3.38	0.18 (−0.47, 0.84)
list the various types of publications?	2.76	3.10	0.34 (−0.29, 0.96)
describe the elements of a manuscript?	3.00	3.57	0.57 (−0.09, 1.23)
describe an effective abstract?	2.88	3.10	0.22 (−0.42, 0.85)
list resources for improving one’s writing skills?	2.44	2.90	0.46 (−0.17, 1.10)
describe the need for transparency and its impact on the research process?	3.00	3.48	0.48 (−0.08, 1.04)
describe the need for rigor and reproducibility and their impact on the research process?	3.12	3.48	0.36 (−0.28, 0.99)
identify approaches to enhance the transparency, rigor, and reproducibility of your research project?	2.68	3.29	0.61 (0.02, 1.19)*
list resources for study design and data analysis?	2.29	2.86	0.57 (0.00, 1.13)*
list venues for presenting research projects?	2.28	3.00	0.72 (0.07, 1.38)*
**Interest in Research**			
How much do you enjoy conducting research?^	2.36	1.95	−0.41 (−0.97, 0.15)
I am interested in pursuing research opportunities DURING residency.^**†**^	3.52	3.00	−0.52 (−1.16, 0.12)
I am interested in pursuing research opportunities AFTER residency.^**†**^	2.96	2.39	−0.58 (−1.29. 0.13)
**Perception in Importance** ^**†**^			
Research experience is important to my residency training.	3.44	3.24	−0.20 (−0.76, 0.35)

The scale ranged from 1 (Not confident) to 5 (100% confident).

^ Responses ranged from 1 (Not at all) to 5 (It is one of the greatest pleasures of my life).

† Responses ranged from 1 (Strongly disagree) to 5 (Strongly agree). Mean differences with their 95% Confidence Interval (95%CI) performed with Student *T*-test.

* *P* value < 0.05.

## Discussion

Our curriculum demonstrated a standardized, reproducible approach to improve the scholarship program for emergency medicine residents. This curriculum succeeded in the production of peer-reviewed scholarly products that meet the ACGME scholarly activity requirement, provided residents with immersion in a research experience, and gave them tangible products to bolster their post-residency applications, all by the end of their first year of residency. Senior resident leaders gained a first author, conference abstract presentation and peer-reviewed publication, and peer leadership experience. Further, this program provided a feasible approach to meet the suggested framework for high-quality scholarship [6].

This novel approach to resident research allowed early research immersion in residency training to provide both project-based learning and to facilitate exposure for residents who may consider future research training. This approach may be generalizable to other residency programs, regardless of clinical specialty. The use of chart review is one potential study design that is feasible and leverages the cohort of residents who participated. By conceptualizing and refining the project prior to the arrival of the first-year residents, this preparatory work greatly facilitated participation and timely completion of the project within the year but may have had the unintended consequence of diminishing enthusiasm for the study topic. We also found that variable resident attendance due to shift times and off-service rotations that might diminish engagement could be mitigated by recording in-person meetings and lectures for later review.

The pre- and post-survey data suggest our intervention improved resident confidence in research methodology across four measures. By increasing confidence in research methodology, residents may be better prepared to critically analyze and apply principles of evidence-based medicine into their daily practice.

There are three important limitations to this approach to resident research curriculum. First, our residency has a highly engaged and robust research division which may limit generalizability to other academic programs. Programs without such research expertise within their department may consider partnering with other departments or institutions. Second, we assessed resident perceptions of confidence in research domains rather than actual competency. Further, this was a small sample size as evidenced by the wide confidence intervals. With completion of future resident cohorts, we will also assess attitudes toward research as a career and the pursuit of research fellowships. Finally, we did not track individual responses before and after the program because we conceptualized this as an anonymous survey, which may limit the identification of individuals with a greater enthusiasm for future research opportunities.

## Conclusion

This novel resident research curriculum developed for an emergency medicine residency program demonstrated a standardized, reproducible, and sustainable approach to provide residents with an immersive research experience. Residents demonstrated increased confidence in their research skills. Further investigation is necessary to examine the impact on future resident research knowledge and willingness to pursue future research training.
